# Germination and Early Development of Three Spontaneous Plant Species Exposed to Nanoceria (*n*CeO_2_) with Different Concentrations and Particle Sizes

**DOI:** 10.3390/nano10122534

**Published:** 2020-12-17

**Authors:** Daniel Lizzi, Alessandro Mattiello, Barbara Piani, Guido Fellet, Alessio Adamiano, Luca Marchiol

**Affiliations:** 1 DI4A—Department of Agriculture, Food, Environment and Animal Sciences, University of Udine, Via delle Scienze 206, 33100 Udine, Italy; lizzi.daniel.1@spes.uniud.it (D.L.); alessandro.mattiello@uniud.it (A.M.); barbara.piani@uniud.it (B.P.); guido.fellet@uniud.it (G.F.); 2 Department of Life Sciences, University of Trieste, Via Licio Giorgieri 10, 34127 Trieste, Italy; 3 Institute of Science and Technology for Ceramics (ISTEC), National Research Council (CNR), Via Granarolo 64, 48018 Faenza, Italy; alessio.adamiano@istec.cnr.it

**Keywords:** nanomaterials, cerium oxide nanoparticles, wild herbs, seed germination, root length

## Abstract

This study aimed to provide insight regarding the influence of Ce oxide nanoparticles (*n*CeO_2_) with different concentrations and two different particle sizes on the germination and root elongation in seedlings of spontaneous terrestrial species. In a bench-scale experiment, seeds of the monocot, *Holcus lanatus* and dicots *Lychnis-flos-cuculi* and *Diplotaxis tenuifolia* were treated with solutions containing *n*CeO_2_ 25 nm and 50 nm in the range 0–2000 mg Ce L^−1^. The results show that *n*CeO_2_ enters within the plant tissues. Even at high concentration, *n*CeO_2_ have positive effects on seed germination and the development of the seedling roots. This study further demonstrated that the particle size had no influence on the germination of *L. flos-cuculi*, while in *H. lanatus* and *D. tenuifolia,* the germination percentage was slightly higher (+10%) for seeds treated with *n*CeO_2_ 25 nm with respect to 50 nm. In summary, the results indicated that *n*CeO_2_ was taken up by germinating seeds, but even at the highest concentrations, they did not have negative effects on plant seedlings. The influence of the different sizes of *n*CeO_2_ on germination and root development was not very strong. It is likely that particle agglomeration and ion dissolution influenced the observed effects.

## 1. Introduction

Nanoscience and nanotechnology are rapidly evolving in different applications having the potential to revolutionize human life. Considerable headways have been made for applications of engineered nanomaterials (ENMs) and nano-enabled products in medicine, energy, electronics, innovative materials and many others [1].

The flip side of nanotechnology is the release in the environment of tons of ENMs [2]. According to the ENMs flow models, soils and waters are the endpoints of such materials [3,4]. However, we still have patchy knowledge regarding the impacts of these materials on biota [5]. Since plant Kingdom represent about 82% of living organisms mass on Earth [6], and their ecological role is of paramount importance to understand the relationships between plants and ENMs. In particular, studying the behavior and fate of ENMs within plants is of great significance for exploring (i) ENMs uptake, translocation and storage in plant tissues, (ii) mechanisms of plant toxicity, and (iii) life cycle risk assessment of ENMs and risks of transfer to the trophic chain.

The early experimental demonstration regarding the negative influence of ENMs in higher plants was carried out not many years ago [7]. Subsequent studies reported physiological and morphological anomalies of plants exposed to nanomaterials [8,9]. Conversely, several studies of positive effects of ENMs applications to crops were reported. This is why applications of nano-enabled products in crop nutrition and protection are under investigation [10,11,12]. The first investigations revealed that the relationships between plants and ENMs are very complex. Up to now, the research has been paid almost exclusively to food crops, while the spontaneous plant species have been almost neglected. Although this was largely justified by the potential risks for ENMs human exposure, the potential negative impact of ENMs on primary producers could have very serious consequences on food webs and ecosystem services [13], and therefore, it should not be deemed less significant.

Experiments carried out on crops demonstrated that the chemical and physical properties of ENMs (e.g., size, shape, structure, composition, concentration, and others), the environmental conditions, the plant species and age contribute to determining the effects on plants [14,15]. It is not advisable to generalize the results on crops to other plants living on natural ecosystems, neither fertilized nor irrigated, and potentially more exposed to ENMs fluxes having a longer life-cycle than crops.

Studies have been conducted to investigate the flow of nanomaterials into aquatic and terrestrial environments. As regards plants, more aquatic [16,17,18,19,20] and wetland species [21,22,23] have been studied than terrestrial ones so far. To the best of our knowledge, *Pinus sylvestris* (L.) and *Quercus robur* (L.) are the only terrestrial wild plant species that have been investigated for exposure to silver nanoparticles (*n*Ag) and cerium oxide nanoparticles (*n*CeO_2_) so far [24].

Investigations on the effects of metal nanoparticles (MeNPs) on plant physiology are based on the assumption that nanomaterials can be absorbed by plants and that the former can subsequently move within the plant tissues while maintaining the nanoform, or that they can release elements in ionic form. Hence, the experiments in this field must be designed in order to verify whether the nanomaterials are taken up by the plant roots or internalized through other pathways such as stomata, leaf cuticle/epidermis, and hydathodes [25].

Given the estimated global production of 100–1000 tons per year, *n*CeO_2_ is among the most widely utilized metal oxide nanoparticle in Europe [26]. For this reason, the Organization for Economic Cooperation and Development (OECD) included *n*CeO_2_ among the nanoparticles to be studied and analyzed for the risk assessment [27]. *n*CeO_2_ could cause several effects on the plant system depending on *n*CeO_2_ particle size, treatment concentration and plant species. Literature reports contradictory results. Positive effects in terms of germination, biomass yield, photosynthesis and nutritional status have been observed on several species [28]. Other papers report a reduction of germination rates, reduction or inhibition of root growth, restrictions of biomass growth, and crop yield [29,30,31].

In this study, we evaluated the influence of *n*CeO_2_ having different concentrations and two particle sizes on the germination and root elongation in seedlings of the spontaneous monocot *Holcus lanatus* (L.), and the dicots *Lychnis flos-cuculi* (L.) and *Diplotaxis tenuifolia* (L.) DC. The plant species have been chosen since they are common and widespread in natural systems, highly competitive and easily adaptable to different ecological conditions. *Holcus lanatus* L. (common velvet grass) is a hairy, tufted, fibrous-rooted and meadow soft perennial grass, growing between 50 and 100 cm tall, belonging to the *Poaceae* family. It has a wide climatic range and occurs over a wide range of soil types and fertility conditions [32]. *Lychnis flos–cuculi* L. (ragged-robin) is an herbaceous perennial plant belonging to the *Caryophyllaceae* family, and it is native and distributed throughout Europe [33]. It is found in open habitats, along roads and in wet meadows and pastures. Finally, *Diplotaxis tenuifolia* L. DC. (perennial wall rocket) is a perennial flowering herbaceous Mediterranean species, but it is native to Europe and Western Asia [34]. It grows in temperate climates and could be found in different habitats, but in particular in ruderal plant associations.

## 2. Materials and Methods

### 2.1. Nanoparticles Characterization

The *n*CeO_2_ with an average particle size of 25 nm and 50 nm, respectively, were purchased from Sigma-Aldrich (St. Louis, MO, USA). *n*CeO_2_ has a density of 7.13 g mL^−1^ at 25 °C and 99.95% purity (81.25% of Ce).

The cerium oxide nanoparticles were suspended in deionized water and sonicated in a water bath for 60 min with a sonication power of 180 watts. The suspensions were characterized for Z–average size and hydrodynamic diameter (Hd), whose distributions were measured by dynamic light scattering (DLS) on a Zetasizer Nano ZS (Malvern Ltd., Worcestershire, UK) and relative polydispersity index (PDI). ζ—potentials at pH 7.0 were quantified by laser Doppler velocimetry as the electrophoretic mobility, using a disposable electrophoretic cell (DTS1061, Malvern Ltd., Worcestershire, UK). The size and average shape were measured with transmission electron microscopy (TEM, FEI Tecnai F20, FEI Company, Eindhoven, The Netherlands).

### 2.2. Experimental Setup

Seeds of *H. lanatus* and *L. flos–cuculi* were purchased from SemeNostrum (Udine, Italy), while seeds of *D. tenuifolia* were provided by Sementi Bruni (Corbetta, Milan, Italy). The experiment was carried out in controlled conditions. 30 seeds were placed into 15 mm Petri dishes containing filter paper soaked with 10 mL of deionized water (control) and 0.2, 2, 20, 200 and 2000 mg mL^−1^ of *n*CeO_2_ 25 nm and *n*CeO_2_ 50 nm suspensions. The suspensions of nanoceria were prepared and sonicated for 10 min to avoid aggregation. The Petri dishes were covered with aluminum paper to avoid light and set at room temperature (25 °C). The duration of the experiment was of two weeks. Each treatment was replicated three times. Germination was calculated as the ratio of germinated seeds out of the total seeds in each Petri dish. Seedlings were photographed, and Image J software [35] was used to measure roots length, which was calculated as the average of measures of all roots that emerged from seeds for each treatment.

### 2.3. Ce concentration in Plant Seedlings

To quantify the total content of Ce inside different plant species, seedlings were washed by agitation with HNO_3_ 0.01 M for 15 min and rinsed with deionized water. The washed seedlings were oven-dried at 60 °C for three days, and 0.3 g of tissues were digested on a microwave oven (MARS Xpress, CEM, Matthews, NC, USA), using 9 mL of HNO_3_ and 1 mL of hydrogen peroxide (H_2_O_2_) in Teflon cylinders at 180 °C. Plant extracts were diluted and filtered with Whatman 0.45 μm PTFE membrane filters. During the ICP–MS analysis, yttrium was the internal standard used for the analysis [36].

### 2.4. Internalization of nCeO_2_ in Plant Tissues

At the end of the germination experiment, the uptake of *n*CeO_2_ by plant seedlings was verified by enzymatic digestion. The digesting enzyme used was Macerozyme R−10 enzyme–pectinase from *Rhizopus* sp. (Sigma-Aldrich Co., St. Louis, MO, USA). The extraction of *n*CeO_2_ from homogenized samples of these species was performed according to Jiménez-Lamana et al. (2016) [37]. In particular, 0.03 g of fresh plant samples were harvested, rinsed with deionized water and homogenized with 8 mL of 2 mM citrate buffer at pH 4.5, using an ultrasonic bath for 5 min. After the homogenization, 2 mL of the enzyme solution (0.05 g of enzyme powder for roots, shoots, leaves and seedlings, dissolved in 2 mL of MilliQ water) was added. The samples were shaken in a water bath at 37 °C for 24 h, and the obtained suspensions were filtered with a 0.45 μm cellulose filters to remove the solid parts of seedlings. The final supernatants were appropriately diluted and analyzed using the single particle inductively coupled plasma mass spectrometer (sp-ICP-MS) NexION 350 (PerkinElmer Waltham, MA, USA).

### 2.5. Data Analysis

Statistical analysis was carried out with three-, two- and one-way analysis of variance (ANOVA). When necessary, data were subjected to logarithmic transformation prior to analysis, which effectively homogenized the variances and produced approximately normal distributions. A posteriori comparison of individual means was performed using Tukey’s test (*p* < 0.05). Differences between treatments for the different measured variables were tested using one-way ANOVA. Data are expressed as mean ± standard deviation (SD). Sp–ICP–MS data on nanoceria size distribution were processed by means of Syngistix Nano Application Module software (PerkinElmer Waltham, MA, USA) and interpolated with polynomial curves.

The *n*CeO_2_ concentration range (0–2000 mg L^−1^) was chosen considering that the large body of literature studies reporting the effects of *n*CeO_2_ on plant physiology used Ce concentrations in the range 1–1000 mg L^−1^ [4,37], while the phytotoxicity test, as recommended by the USEPA approach, used the 2000 mg L^−1^ level [38].

## 3. Results

### 3.1. Characterization of nCeO_2_

**Figure 1:**
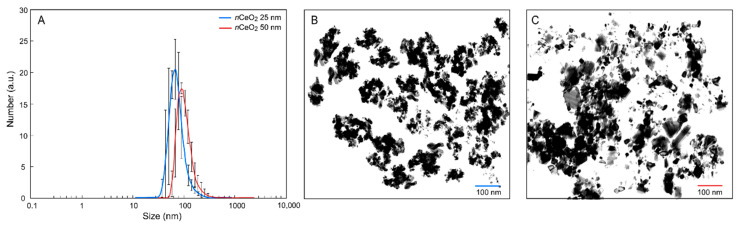
(**A**) Particle size distribution measured by dynamic light scattering (DLS) on suspensions of *n*CeO_2_ 25 nm and 50 nm; (**B**) transmission electron microscopy (TEM) image of *n*CeO_2_ 25 nm; (**C**) TEM *n*CeO_2_ 50 nm.

The results of the physicochemical characterization of *n*CeO_2_ are reported in Table 1 and Figure 1A. In particular, the relative Z—averages are reported in Table 1, together with the relative polydispersity index (PDI) and the ζ—potentials of the particles. The Hd distribution of both the materials is in agreement with the value provided by the supplier. Both *n*CeO_2_ 25 nm and 50 nm exhibit a monodisperse size particle distribution in the nanometric range with relatively low PDI, and the main size peak at 62.0 nm and 91.0 nm, respectively. The relative Z-averages were found to be much larger than these values; this was probably due to the presence of particle aggregates. Since a high net surface charge is typically associated with weak nanoparticle interactions and aggregation, these data are coherent with the higher Z-average detected for sample *n*CeO_2_ 50 nm with respect to *n*CeO_2_ 25 nm [39]. Figure 1B,C reports fields of TEM observation of *n*CeO_2_ 25 and 50 nm suspensions.

### 3.2. nCeO_2_ Plant Internalization

**Figure 2:**
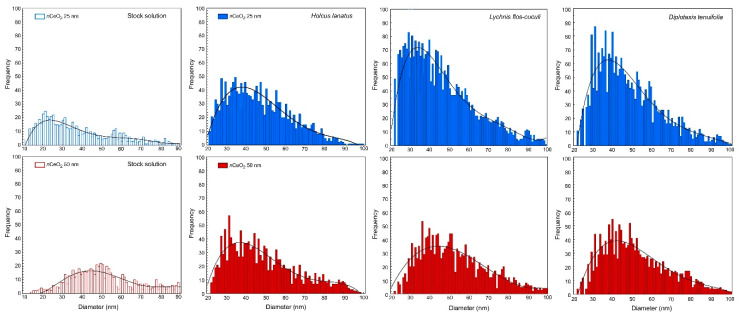
Particle size distributions of, respectively, *n*CeO_2_ 25 nm and *n*CeO_2_ 50 nm stock solutions (open bars) and after enzyme treatment of seedlings of *H. lanatus*, *L. flos-cuculi* and *D. tenuifolia* (closed bars) treated with 20 mg L^−1^ of *n*CeO_2_.

The early step of our study was devoted to verifying the entry of *n*CeO_2_ into plant tissues. Control and treated seedlings of *H. lanatus*, *L. flos-cuculi* and *D. tenuifolia* were subjected to the extraction procedure and further analyzed by sp–ICP–MS. Size distributions of ceria nanoparticles in stock solutions and in the seedlings treated with 20 mg L^−1^ *n*CeO_2_ 25 nm and 50 nm are reported in Figure 2.

As expected, in control seedlings, *n*CeO_2_ was not detected, whereas in all treated species, (i) the presence of internalized *n*CeO_2_ was verified, and (ii) the *n*CeO_2_ have a different size distribution than stock solution suggesting aggregation phenomena between nanoparticles (Figure 2 and Table 2). Data from sp–ICP–MS analysis confirmed that *n*CeO_2_ underwent agglomeration. The increase of the median diameter of *n*CeO_2_ was evident for seedlings treated with *n*CeO_2_ 25 nm, being 41.7 nm the average size of the nanoparticles extracted from seedlings (41 nm in *H. lanatus* and *L. flos-cuculi*, and 43 nm in *D. tenuifolia*). The mean size of particles extracted from seedlings treated with *n*CeO_2_ 50 nm was 47 nm so in good agreement with the treatment (Figure 2 and Table 2).

The sp–ICP–MS results also show that the most frequent size of nanoparticles taken up by plants is similar for monocotyledons and dicotyledons for *n*CeO_2_ 50 nm, whereas, for 25 nm treatments, the most frequent diameter is smaller in *L. flos-cuculi* and *D. tenuifolia* (respectively 31 and 35 nm) than *H. lanatus* (40 nm) (Figure 2 and Table 2). Regarding this aspect, some authors evidenced a size-dependent uptake and translocation of *n*CeO_2_ in plants. In particular, *n*CeO_2_ having a diameter smaller than 50 nm are present in all plant tissues and pass from roots to the aerial parts without dissolution and transformation.

### 3.3. Seed Germination and Root Length

A three-way ANOVA was run in order to have a general view regarding the effects of plant species, *n*CeO_2_ size and Ce concentration on (i) percentage of germination, (ii) root length and (iii) Ce concentration in plant tissues. There were significant three-way interactions for percentage of germination (*p* = 0.0463 *) and root length (*p* = 0.0000 ***) (Table 3). Subsequently, the statistical analysis with two-way ANOVA within the species continued.

**Figure 3:**
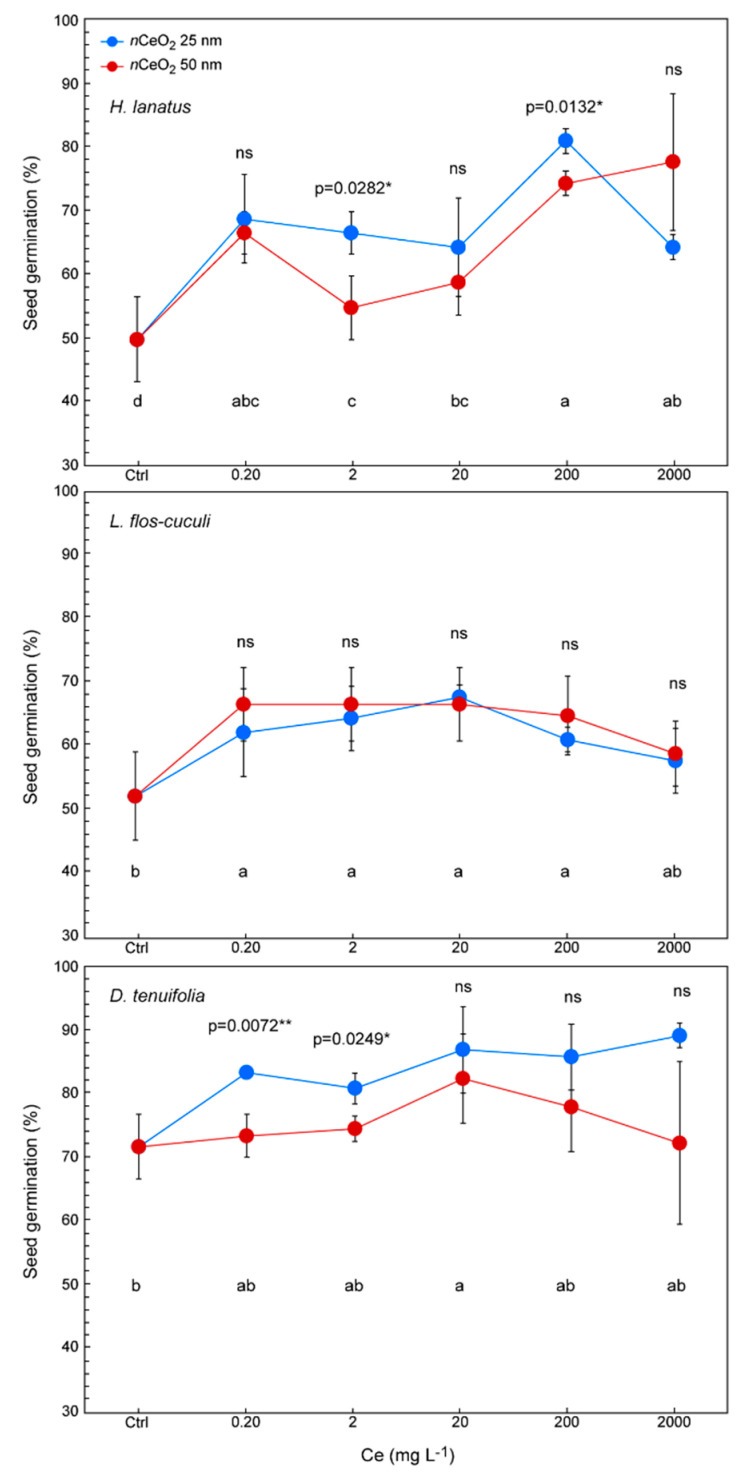
Percentage of seed germination in *H. lanatus*, *L. flos-cuculi* and *D. tenuifolia*, grown in Petri dishes and treated with solutions of *n*CeO_2_ 25 nm and *n*CeO_2_ 50 nm at 0, 2, 20, 200 and 2000 mg L^−1^, respectively. The values are mean ± SD (standard deviation) of 3 replicates. Statistical significance of the treatments for each Ce concentration is reported: (i) figure upper part→comparison between *n*CeO_2_ 25 nm and 50 nm; (ii) figure lower part→comparison between all treatments. Different letters indicate statistical differences. ns = not significant, * *p* ≤ 0.05, ** *p* ≤ 0.01.

As shown in Figure 3, treatments improve the germination percentage in all the three species if compared with controls. Indeed, germination increases more than 20% in several treatments in *H. lanatus*, 15% in *L. flos-cuculi* and 10% in *D. tenuifolia*, with respect to the control.

The evaluation of the effects induced by *n*CeO_2_ of different sizes is the main objective of this study. Actually, we have not verified a clear trend related to the *n*CeO_2_ size. In fact, the nanoparticle dimensions had no influence on the germination of *L. flos-cuculi*, while in both the other species, in some cases, they did. We observed that the germination percentage is higher for seeds treated with *n*CeO_2_ 25 nm (about +10%) compared with 50 nm. (Figure 3). A statistically significant difference was found in *H. lanatus* at 2 and 200 mg L^−1^ (respectively, *p* = 0.0282 * and *p* = 0.0132 *) and *D. tenuifolia* at the at 0.2 and 2 mg L^−1^ (respectively, *p* = 0.0072 ** and *p* = 0.0249 *) (Figure 3). It is quite likely that in this species, the influence of the size of *n*CeO_2_ on germination could have been observed even at the highest concentration, but the high variability of the data influenced the response of the statistics (*p* = 0.0866) (Figure 3).

**Figure 4:**
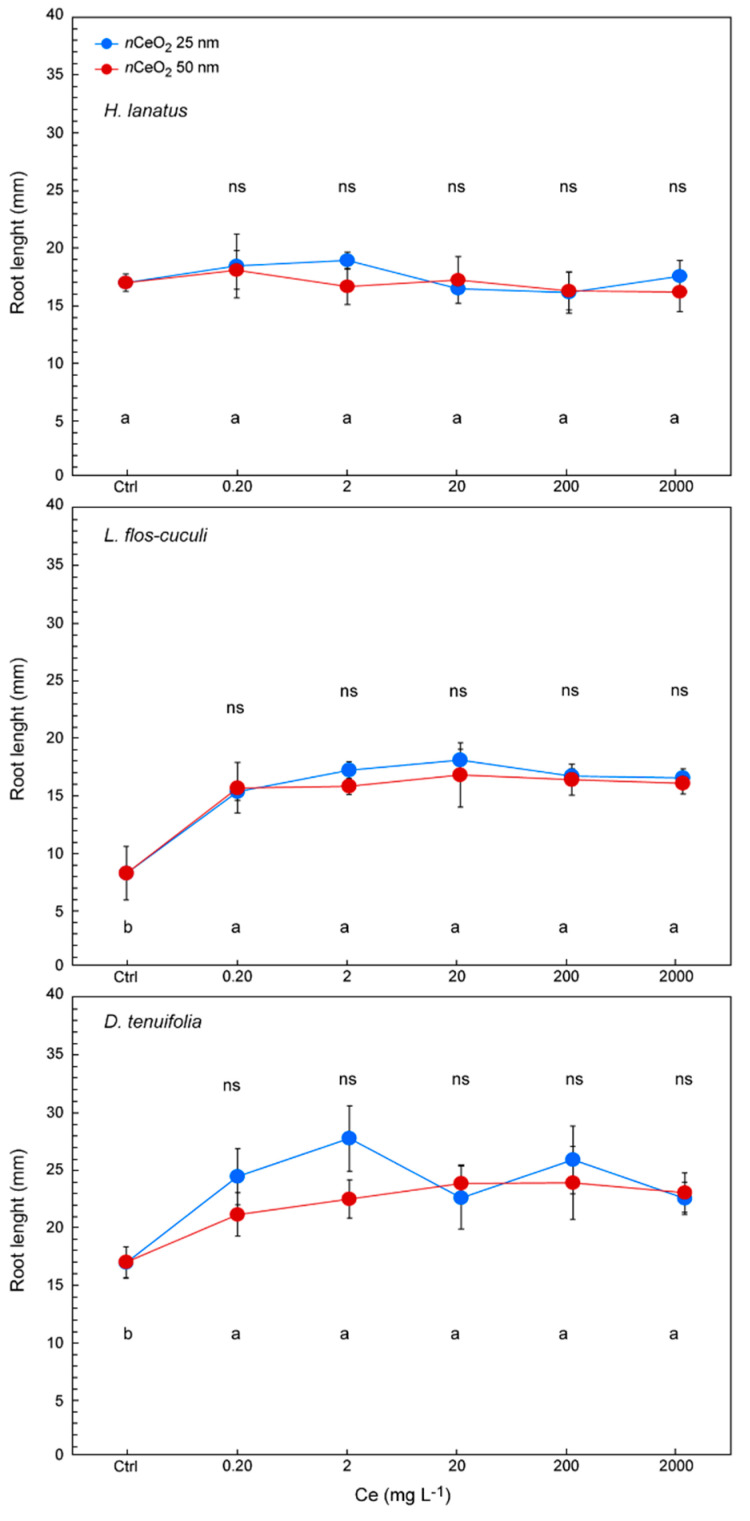
Root length in seedlings of *H. lanatus*, *L. flos-cuculi* and *D. tenuifolia*, grown in Petri dishes and treated with solutions of *n*CeO_2_ 25 nm and *n*CeO_2_ 50 nm at 0, 2, 20, 200 and 2000 mg L^−1^, respectively. The values are mean ± SD (standard deviation) of 3 replicates. Statistical significance of the treatments for each Ce concentration is reported: (i) figure upper part→comparison between *n*CeO_2_ 25 nm and 50 nm; (ii) figure lower part→comparison between all treatments. Different letters indicate statistical differences. ns = not significant, * *p* ≤ 0.05, ** *p* ≤ 0.01.

Previously we demonstrated some relationships between the germination process and *n*CeO_2_ size. Similar observations were carried out on the seedling root length (Figure 4).

In this case, the results demonstrate that, regardless of the Ce concentration, root length was not influenced by the *n*CeO_2_ size. However, treatments stimulate the root growth in all three species, with a clear increase of length, in particular in *L. flos-cuculi* and *D. tenuifo*lia, if compared with control seedlings (Figure 4). Some differences in response to treatments are species-specific. The *n*CeO_2_ of both sizes do not have any stimulating effect on the length of the roots of *H. lanatus*, which resulted in insensitivity to the treatments even at higher concentrations. On the other hand, we observed an increase in root length in treated seedlings of the other species. This effect was particularly intense in *L. flos-cuculi,* where the length of the roots of treated seedlings on average has almost doubled (+90.1%) compared to the control (14.7 mm and 7.73 mm, respectively). The stimulating effect of *n*CeO_2_ demonstrated in *D. tenuifolia* is much less powerful but remarkable, where we found a 34% increase in root length compared to the control (22.8 mm and 16.9 mm, respectively).

### 3.4. Ce Concentration in Plant Seedlings

**Figure 5:**
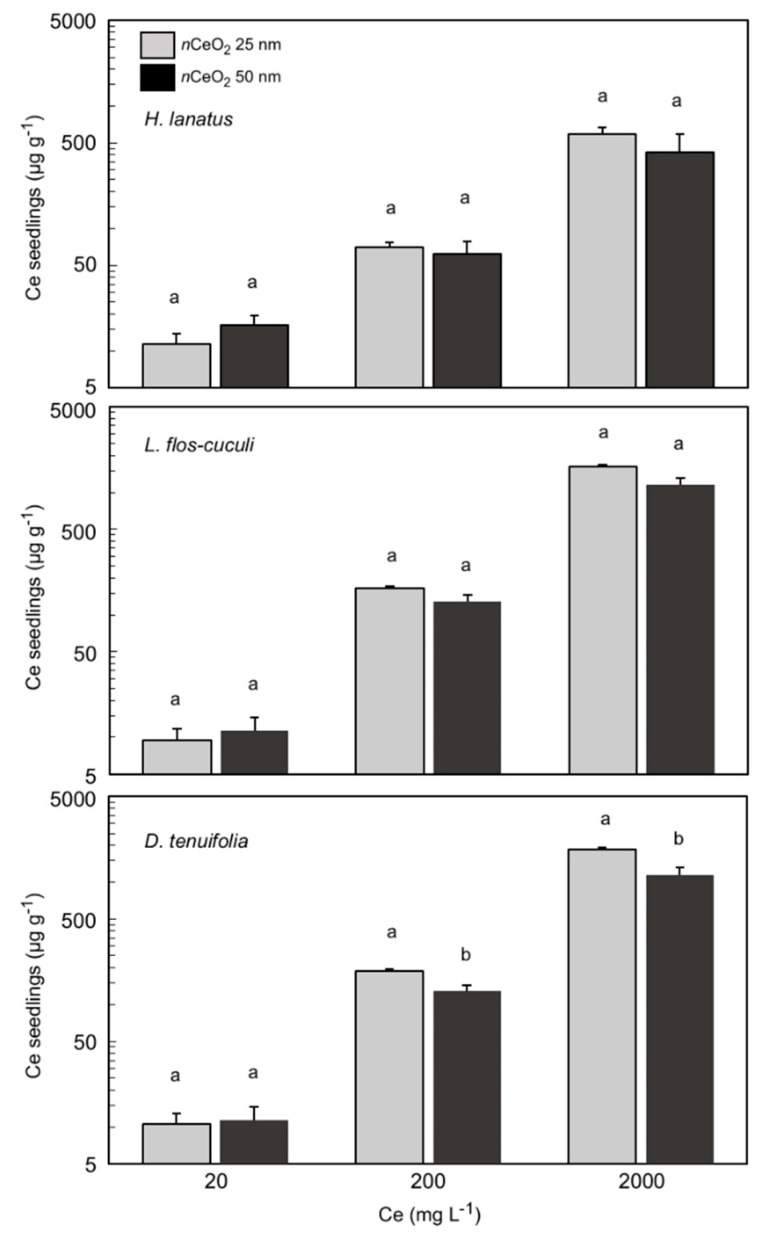
Ce concentration in seedlings of *H. lanatus*, *L. flos-cuculi* and *D. tenuifolia*, grown in Petri dishes and treated with solutions of *n*CeO_2_ 25 nm and *n*CeO_2_ 50 nm at 20, 200 and 2000 mg L^−1^, respectively. The values are mean ± SD (standard deviation) of 3 replicates. Statistical significance of the treatments (ANOVA *p* value) for each Ce concentration is reported. Different letters indicate statistical differences (*p* ≤ 0.05).

To quantify the total content of Ce that was taken up by seedlings in the three plant species, we used an ICP–MS after the acid digestion of the samples. The elaborated data with the total concentration of Ce are presented in Figure 5.

The concentration of total Ce in seedling tissues of *H. lanatus*, *L. flos-cuculi* and *D. tenuifolia* shows a different magnitude of accumulation according to the treatments. In fact, a statistically significant effect of treatments (*p* < 0.05) was verified for all the species. As already reported in Table 2, the interaction “species x Ce concentration” was highly statistically significant (*p* = 0.0000 ***). With regard to the *n*CeO_2_ size, we observed that the seedlings treated with the smaller *n*CeO_2_ reveal a higher concentration of Ce in their tissues than the ones treated with the 50 nm nanoparticles. This occurred in particular in *L. flos-cuculi* and *D. tenuifolia* and at the two highest concentrations of treatments, but not in *H. lanatus*. In *L. flos-cuculi*, the total content of Ce corresponds to 165 and 128 mg kg^−1^ DW at 200 mg L^−1^; 1616 and 1151 mg kg^−1^ DW at 2000 mg L^−1^ (*p* = 0.0134 *), respectively at 25 nm and 50 nm. In *D. tenuifolia* we detected 189 and 114 mg kg^−1^ DW at 200 mg L^−1^; 1841 and 1305 mg kg^−1^ DW at 2000 mg L^−1^ (*p* = 0.0465 *), respectively at 25 nm and 50 nm (Figure 5).

The size of the nanoparticles in our study was 25 nm and 50 nm. By looking at Figure 2, it turns out that the stock solutions of the two nominal sizes are actually a mixture of nanoparticles of different dimensions, with 25 nm and 50 nm being the dimensions among the ones having the highest frequencies. This makes one conclude that both the dispersions contain nanoparticles that can potentially enter the plant roots. There are two main factors, among others, that can influence the uptake and translocation of the nanoparticles: (i) the size of the pores in the cell membrane; (ii) the tendency of the nanoparticles to aggregate due to chemical interactions. Given the fact that the dimension of the nanoparticles at a given shape determines the surface to volume ratio, this can affect the entity of such aggregation (the smaller the dimension, the more likely the aggregation).

Tables S1 and S2 report the calculations of the *n*CeO_2_ 25 nm and 50 nm ratios based on the assumptions that all the nanoparticles are spherical and equal in size (25 nm or 50 nm) in the dispersions as well as inside the seedlings and no aggregations occur. The theoretical ratio (Table S1) refers to an ideal scenario for which an equal mass of nanoparticles is taken up by both the plants exposed to the *n*CeO_2_ 25 nm and the *n*CeO_2_ 50 nm; the theoretical ratio is hence obtained by calculating the number of nanoparticles at a given size (25 nm or 50 nm) as the mathematical division of the total mass of Ce in the plant by the mass of a single nanoparticle. The observed ratio is calculated following the same procedure and assumptions, but considering the experimental mean Ce mass measured in planta for the different treatments (Table S2).

## 4. Discussion

The previously published studies carried out in controlled conditions, such as Petri dishes and hydroponics, reported that the toxicity of nanomaterials in the initial development stages of plant growth could be due to physicochemical properties, as well as particle size and shape [40,41]. In general, MeNPs show early negative consequences on the development stages of crops and this observation is confirmed in some publications [7,8,42,43].

Literature papers suggest that *n*CeO_2_ generally enters plants through root uptake and may cause several effects on the early stages of plant development, such as reducing or increasing germination rates and improving, reducing or inhibiting radical growth [44,45]. When germinating seeds are exposed to nanoparticles, different effects could be verified, basically depending on the plant species and particle size and concentration [8].

It was demonstrated that *n*CeO_2_ having a diameter comprised in the range 50–100 nm are taken up by roots, but they hardly move towards the aerial plant fractions, while *n*CeO_2_ larger than 100 nm is not absorbed by roots [46,47]. We observed that the formation of particle agglomerates concerns, in particular *n*CeO_2_ 25 nm. It is very likely that this was due to the higher specific surface than *n*CeO_2_ 50 nm. At the same time, sp–ICP–MS analysis showed the largest number of peaks in all seedlings treated with *n*CeO_2_ 25. Confirming previous literature findings [48], this suggests that the smaller particle size has the ability to enter into the roots more easily than 50 nm. Combining the previous evidence, we hypothesize that the *n*CeO_2_ 25 nm agglomeration occurred inside the seedling tissues.

According to Layet et al. (2017) [49], we demonstrate that the two dicotyledons take up more *n*CeO_2_ than *H. lanatus*. Since seedlings of the different species growing in the same conditions, it is likely that the uptake and translocation of *n*CeO_2_ are influenced by species-specific physiological traits. The ability of *n*CeO_2_ uptake by roots and subsequent translocation to the other parts of plants was already demonstrated in crop species [50,51,52]. We observed a similar particle size distribution for *n*CeO_2_ 25 nm in *L. flos-cuculi* and *D. tenuifolia*. At the same time, no relevant changes were observed for *n*CeO_2_ 50 nm.

We recorded a negligible dissolved concentration of Ce ions in all samples, indicating that *n*CeO_2_ did not undergo dissolution after being absorbed by roots. On the other hand, we observed that small signals of dissolved forms of Ce correspond to the presence of bigger nanoparticles (50 nm), as previously reported [52,53]. Hence, it is likely that *n*CeO_2_ 25 nm after being taken up by the seedling roots move through the vascular system forming aggregates. This process has been explained by the attraction between nanoparticles caused by van der Waals forces or chemical bonds [54,55]. However, since this also occurs within the plant tissues, it is still unclear whether and how species-specific factors can influence this process.

A large number of studies highlight that in some plant species, particle agglomeration happens before the passage from roots to the other parts of seedlings [42,56,57,58]. This statement could be justified by the plausible hypothesis that MeNPs pass through the apoplastic pathway [59] or cause the destruction of some cell walls, and in so doing, they pass through the enlarged pores [60]. However, nanoparticles could enter the vascular system where the Casparian strip is not formed [61,62] or through the lateral root junction [25,61,63,64].

Our data also indicated that the treatments with *n*CeO_2_ of different size influenced the size distribution of nanoparticles within the plant tissues. This could be due to the smaller size of the materials that lead to an exponential increase in surface area relative to volume in contact with roots. We can conclude that the *n*CeO_2_ entered inside the seedlings, and therefore, the results that will be described later are reasonably influenced by the experimental treatments. With regard to *n*CeO_2_ aggregation, in this study, we did not develop further observations.

With regard to the observed stimulating effect on germination of *n*CeO_2_, it must be said that on this point, the literature reports conflicting data. Low toxicity and reduction of seed germination were observed on *Lycopersicum esculentum* and *Zea may*s [44] and *Glycine max* [65], whereas germination of *Hordeum vulgare* was indifferent up to 2000 mg L^−1^ *n*CeO_2_ [66]. As a matter of principle, a direct comparison of data from different experiments is difficult. However, if we were looking to draw a conclusion from available literature data on this point, we can say that *n*CeO_2_ does not cause acute toxicity in the early stages of plant development.

Leaving the nanoscale, it can be confirmed that Ce influence positively seeds germination. As for other rare earth elements, it has been suggested that Ce may have a positive effect by enhancing the effects of phytohormones on germinating seeds [67,68]. In addition, it seems that eventually, monocots species are more tolerant of Ce than dicot ones [69].

Contradictory literature evidence regards the influence of *n*CeO_2_ on root development in different stages of plant growth. Positive effects on root growth were observed in *Zea mays*, *Cucumis sativus* and *Lactuca sativa* [44,59]. As occurred in our species, very high tolerance to *n*CeO_2_ was reported for *Cucumis sativus*, *Brassica oleracea*, *Brassica napus* and *Raphanus sativus*, whose root growth resulted not affected up to *n*CeO_2_ 2000 mg L^−1^. Oppositely, a slowed root development in treated *Medicago sativa* and *Lycopersicum esculentum* was reported [44].

As regards the Ce concentration (or mass per volume) in the seedlings exposed to the two dispersions, it is possible to conclude that the amount of Ce is greater for *n*CeO_2_ 25 nm. Actually, this is not very informative in terms of the number of nanoparticles inside the plants if we consider the fact that the two dispersions had the same quantity of Ce in mass but different amounts in terms of nanoparticles. If we assume that the nanoparticles of the two dispersions are of the same shape and that the frequency of the dimension is 100% for both the dimensional class (25 and 50 nm), then we can conclude that the seedlings exposed to the *n*CeO_2_ 25 nm were in fact exposed to 8 times the number of nanoparticles when compared to the 50 nm dispersion, since the volume goes with the cubic pattern.

Following this reasoning and assuming that the cell membrane pore size is not limiting the entrance of the nanoparticles because the mean size is greater than the sizes of the nanoparticles [70,71], it can be shown that the ratio between the number of nanoparticles taken up by the plant, calculated from the mass, is not in a ratio 1 to 8 as it would be if the two types of nanoparticles were taken up at the same level, but it is 1 to a greater number. This can lead us to speculate that the number of 50 nm nanoparticles inside the seedlings is lower than expected, and this could be due to the aggregation of the nanoparticles outside the plants that limited the entrance of the 50 nm nanoparticles more than the 25 nm nanoparticles despite their higher tendency to form clusters. On the other side, we may assume that the pores of the cell membrane act as filters and so explain why the *n*CeO_2_ 50 nm reached a lower concentration in the seedlings. Of course, this is based on some assumptions that are not likely to occur in real conditions (the shape and size of the nanoparticle are far from being homogeneous and aggregation occurs). In addition, in more complex conditions, such as at field condition or in experiments that use real soil, the strategies that plants use to absorb nutrients from the substrate can influence, for instance, the solubility of the nanoparticles (e.g., by acidification of the rhizosphere which may change the solubility of the different chemical forms of Ce).

## 5. Conclusions

Under our experimental conditions, the presence of *n*CeO_2_—even at high concentrations—did not cause negative effects on *H. lanatus*, *L. flos-cuculi* and *D. tenuifolia*. On the contrary, *n*CeO_2_ had a stimulating effect in the early stages of development of the plants. The plants’ response with respect to the different *n*CeO_2_ sizes has not been very evident. This aspect requires important insights that must take into account the aggregation/dissolution dynamics of *n*CeO_2_ and the forms of Ce taken-up by plants, as well as the fate of the *n*CeO_2_ assimilated by plants. Our results are quite in accordance with the literature, in which—it must be remembered—there are still rather conflicting results, obtained to a very large extent by observing crop species.

Finally, the knowledge of the effects of the exposure of plants to ENM is limited. It is acknowledged that the flux of ENM in the ecosystem involves the primary producers. That is the reason why it is important to focus the research on this field as well as to develop new methods of investigation suitable to non-food species.

As previously reported, the number of works dedicated to the study of the effects resulting from the exposure of spontaneous terrestrial species to ENMs is very low. This constitutes “per se” the major novelty element of this paper. Our observations were made during the early stages of vegetative development. It will be necessary to extend the study to evaluate the effects of the treatments over the whole plant life-cycle. It will be equally important to compare the responses of plants with respect to single and repeated treatments over time.

## Figures and Tables

**Table 1:** Z-average size, relative polydispersity index (PDI) and ζ—potentials of *n*CeO_2_ 25 nm and 50 nm.

Material	Z-Average	PDI	ζ-Potential
	(nm)		(mV)
*n*CeO_2_ 25 nm	126.7 ± 1.0	0.17 ± 0.01	39.2 ± 1.1
*n*CeO_2_ 50 nm	205.7 ± 1.0	0.25 ± 0.02	24.1 ± 0.8

**Table 2:** Most frequent size, mean size and number of peaks determined by sp–ICP–MS analysis after enzyme treatment of seedlings of *H. lanatus*, *L. flos-cuculi* and *D. tenuifolia* treated with 20 mg L^−1^ of *n*CeO_2_ 25 nm and 50 nm.

Treatment	Species	Most Frequent Size (nm)	Mean Size (nm)	N. of Peaks (n)	Dissolved Ce (ppb)
*n*CeO_2_ 25 nm	*H. lanatus*	40	41	1632	0.12
	*D. tenuifolia*	35	43	2630	0.08
	*L. flos-cuculi*	31	41	3079	0.03
*n*CeO_2_ 50 nm	*H. lanatus*	41	44	1388	0.22
	*D. tenuifolia*	40	48	1871	0.20
	*L. flos-cuculi*	41	49	1842	0.21

**Table 3:** Three-way ANOVA *p* values for the main effects of plant species, *n*CeO_2_ size and Ce concentrations and their interactions on the percentage of germination, seedling root length and Ce concentration in seedling of *H. lanatus*, *L. flos-cuculi* and *D. tenuifolia*.

Effect	% Germination	Root Elongation	Ce Concentration in Seedlings
Species	0.0000 ***	0.0000 ***	0.0000 ***
*n*CeO_2_ size	0.0000 ***	0.0000 ***	0.0000 ***
Ce concentration	0.0161 *	0.0000 ***	0.0000 ***
Species × *n*CeO_2_ size	0.0000 ***	0.0000 ***	0.0000 ***
Species × Ce concentration	0.0115 *	0.0000 ***	0.0000 ***
*n*CeO_2_ size × Ce concentration	0.7171 ns	0.0000 ***	0.0895 ns
Species × *n*CeO_2_ size × Ce concentration	0.0463 *	0.0000 ***	0.0848 ns

ns: not significant at *p* ≤ 0.05; *, ** and *** indicate significance at *p* ≤ 0.05, *p* ≤ 0.01 and *p* ≤ 0.001.

## Supplementary Materials

The following are available online at https://www.mdpi.com/2079-4991/10/12/2534/s1, Table S1: Theoretical ratio calculated at a hypothetical equal mass of nanoparticles uptaken by plants exposed to nCeO_2_ 25 nm and 50 nm. The ratio is calculated by dividing the number of *n*CeO_2_ 25 nm by the number of *n*CeO_2_ 50 nm; both numbers are calculated dividing the mass by the estimated the mass of a single nanoparticle (g/g). Table S2: The observed ratio calculated at the measured mean Ce uptake by the plants exposed to the two *n*CeO_2_ 25 nm and 50 nm for the treatments 200 and 2000 ppm and the two species *L. flos-cuculi* and *D. tenuifolia*. The ratio is calculated by dividing the number of *n*CeO_2_ 25 nm by the number of *n*CeO_2_ 50 nm; both numbers are calculated dividing the mass of Ce derived from the measured mean Ce by the estimated the mass of a single nanoparticle (g/g).

## References

[1] Bainbridge W.S., Roco M.C. (2016). Science and technology convergence: With emphasis for nanotechnology-inspired convergence. J. Nanopart. Res..

[2] Mortimer M., Holden P.A., Marmiroli N., White J., Song J. (2019). Fate of engineered nanomaterials in natural environments and impacts on ecosystems. Exposure to Engineered Nanomaterials in the Environment.

[3] Keller A.A., McFerran S., Lazareva A., Suh S. (2013). Global life cycle releases of engineered nanomaterials. J. Nanopart. Res..

[4] Holden P.A., Gardea-Torresdey J.L., Klaessig F., Turco R.F., Mortimer M., Hund-Rinke K., Cohen Hubal E.A., Avery D., Barceló D., Behra R. (2016). Considerations of environmentally relevant test conditions for improved evaluation of ecological hazards of engineered nanomaterials. Environ. Sci. Technol..

[5] Reddy Pullagurala V.L., Adisa I.O., Rawat S., White J.C., Zuverza-Mena N., Hernandez-Viezcas J.A., Peralta-Videa J.R., Gardea-Torresdey J.L., Marmiroli N., White J., Song J. (2019). Fate of engineered nanomaterials in agroenvironments and impacts on agroecosystems. Exposure to Engineered Nanomaterials in the Environment.

[6] Bar-On Y.M., Phillips R., Milo R. (2018). The biomass distribution on Earth. Proc. Natl. Acad. Sci. USA.

[7] Priester J.H., Ge Y., Mielke R.E., Horst A.M., Moritz S.C., Espinosa K., Gelb J., Walker S.L., Nisbet R.M., An Y.J. (2012). Soybean susceptibility to manufactured nanomaterials with evidence for food quality and soil fertility interruption. Proc. Natl. Acad. Sci. USA.

[8] Miralles P., Church T.L., Harris A.T. (2012). Toxicity, uptake, and translocation of engineered nanomaterials in vascular plants. Environ. Sci. Technol..

[9] Zuverza-Mena N., Martínez-Fernández D., Du W., Hernàndez-Viezcas J.A., Bonilla-Bird N., López-Moreno M.L., Komárek M., Peralta-Videa J.R., Gardea-Torresdey J.L. (2017). Exposure of engineered nanomaterials to plants: Insights into the physiological and biochemical responses. A review. Plant Physiol. Biochem..

[10] Lowry G.V., Avellan A., Gilbertson L.M. (2019). Opportunities and challenges for nanotechnology in the agri-tech revolution. Nat. Nanotechnol..

[11] Kah M., Tufenkji N., White J.C. (2019). Nano-enabled strategies to enhance crop nutrition and protection. Nat. Nanotechnol..

[12] Marchiol L., Iafisco M., Fellet G., Adamiano A. (2020). Nanotechnology support the next agricultural revolution: Perspectives to enhancement of nutrient use efficiency. Adv. Agron..

[13] Hawthorne J., De la Torre Roche R., Xing B., Newman L.A., Ma X., Majumdar S., Gardea-Torresdey J.G., White J.C. (2014). Particle-size dependent accumulation and trophic transfer of cerium oxide through a terrestrial food chain. Environ. Sci. Technol..

[14] Du W., Xu Y., Yin Y., Ji R., Guo H. (2018). Risk assessment of engineered nanoparticles and other contaminants in terrestrial plants. Curr. Opin. Environ. Sci. Health.

[15] Spielman-Sun E., Avellan A., Bland G., Tappero R.V., Acerbo A.S., Unrine J.M., Giraldo J.P., Lowry G.V. (2019). Nanoparticle surface charge influences translocation and leaf distribution in vascular plants with contrasting anatomy. Environ. Sci. Nano.

[16] Asztemborska M., Bembenek M., Jakubiak M., Stęborowski R., Bystrzejewska-Piotrowska G. (2018). The effect of nanoparticles with sorption capacity on the bioaccumulation of divalent ions by aquatic plants. Int. J. Environ. Res..

[17] Ding Y., Bai X., Ye Z., Ma L., Liang L. (2019). Toxicological responses of Fe_3_O_4_ nanoparticles on *Eichhornia crassipes* and associated plant transportation. Sci. Total Environ..

[18] Movafeghi A., Khataee A., Abedi M., Tarrahi R., Dadpour M., Vafaei F. (2018). Effects of TiO_2_ nanoparticles on the aquatic plant *Spirodela polyrrhiza*: Evaluation of growth parameters, pigment contents and antioxidant enzyme activities. J. Environ. Sci..

[19] Ekperusia A.O., Sikokic F.D., Nwachukwud E.O. (2019). Application of common duckweed (*Lemna minor*) in phytoremediation of chemicals in the environment: State and future perspective. Chemosphere.

[20] Geitner N.K., Cooper J.L., Avellan A., Castellon B.T., Perrotta B.G., Bossa N., Simonin M., Anderson S.M., Inoue S., Hochella M.F. (2018). Size-based differential transport, uptake, and mass distribution of ceria (CeO_2_) nanoparticles in wetland mesocosms. Environ. Sci. Technol..

[21] Yin L., Colman B.P., McGill B.M., Wright J.P., Bernhardt E.S. (2012). Effects of silver nanoparticle exposure on germination and early growth of eleven wetland plants. PLoS ONE.

[22] Jacob D.L., Borchardt J.D., Navaratnam L., Otte M.L., Bezbaruah A.N. (2013). Uptake and translocation of Ti nanoparticles in crops and wetland plants. Int. J. Phytoremediation.

[23] Song U., Lee S. (2016). Phytotoxicity and accumulation of zinc oxide nanoparticles on the aquatic plants *Hydrilla verticillata* and *Phragmites australis*: Leaf-type-dependent responses. Environ. Sci. Pollut. Res..

[24] Aleksandrowicz-Trzcińska M., Bederska-Błaszczyk M., Szaniawski A., Olchowik J., Studnicki M. (2019). The effects of copper and silver nanoparticles on container-grown Scots pine (*Pinus sylvestris* L.) and Pedunculate oak (*Quercus robur* L.) seedlings. Forests.

[25] Dietz K.J., Herth S. (2011). Plant nanotoxicology. Trends Plant Sci..

[26] Sun T.Y., Gottschalk F., Hungerbuhler K., Nowack B. (2014). Comprehensive probabilistic modelling of environmental emissions of engineered nanomaterials. Environ. Pollut..

[27] OECD (2008). List of manufactured nanomaterials and list of endpoints for phase one of the OECD testing programme. Safety of Manufactured Nanomaterials No. 6.

[28] Ramirez-Olvera S.M., Trejo-Téllez L.I., García-Morales S., Pérez-Sato J.R., Gomez-Merino F.C. (2018). Cerium enhances germination and shoot growth, and alters mineral nutrient concentration in rice. PLoS ONE.

[29] Lizzi D., Mattiello A., Marchiol L., Tripathi D.K., Ahmad P., Sharma S., Chauhan D. (2017). Impacts of Cerium Oxide Nanoparticles (*n*CeO_2_) on Crop Plants: A Concentric Overview. Nanomaterials in Plants, Algae and Micro-Organisms. Concepts and Controversies.

[30] Skiba E., Wolf W.M. (2019). Cerium oxide nanoparticles affect heavy metals uptake by Pea in a divergent way than their ionic and bulk counterparts. Water Air Soil Pollut..

[31] Adisa I.O., Rawat S., Reddy Pullagurala V.L., Dimkpa C.O., Elmer W.E., White J.C., Hernandez-Viezcas J.A., Peralta-Videa J.R., Gardea-Torresdey J.L. (2020). Nutritional status of tomato (*Solanum lycopersicum*) fruit grown in Fusarium-infested soil: Impact of cerium oxide nanoparticles. J. Agric. Food Chem..

[32] Thompson J.D., Turkington R. (1988). The biology of Canadian weeds—*Holcus lanatus* L.. Can. J. Plant Sci..

[33] Jalas J., Suominen J. (1998). Atlas Florae Europaeae (AFE)—Distribution of vascular plants in Europe 7 (Caryophyllaceae (Silenioideae)).

[34] Hall M.K.D., Jobling J.J., Rogers G.S. (2012). Some perspectives on rocket as a vegetable crop: A review. Veg. Crops Res. Bull..

[35] Schneider C., Rasband W., Eliceiri K. (2012). NIH Image to ImageJ: 25 years of image analysis. Nat. Methods.

[36] Packer A.P., Lariviere D., Li C., Chen M., Fawcett A., Nielsen K., Mattson K., Chatt A., Scriver C., Erhardt L.S. (2007). Validation of an inductively coupled plasma mass spectrometry (ICP-MS) method for the determination of cerium, strontium and titanium in ceramic materials used in radiological dispersal devices (RDDs). Anal. Chim. Acta.

[37] Jiménez-Lamana J., Wojcieszek J., Jakubiak M., Asztemborska M., Szpunar J. (2016). Single particle ICP-MS characterization of platinum nanoparticles uptake and bioaccumulation by *Lepidium sativum* and *Sinapis alba* plants. J. Anal. At. Spectrom..

[38] Zhang W., Yu Z., Rao P., Lo I.M.C. (2019). Uptake and toxicity studies of magnetic TiO_2_-based nanophotocatalyst in *Arabidopsis thaliana*. Chemosphere.

[39] Mukherjee B., Weaver J.W. (2010). Aggregation and charge behavior of metallic and nonmetallic nanoparticles in the presence of competing similarly-charged inorganic ions. Environ. Sci. Technol..

[40] Yang L., Watts D.J. (2005). Particle surface characteristics may play an important role in phytotoxicity of alumina nanoparticles. Toxicol. Lett..

[41] Lin D.H., Xing B.S. (2007). Phytotoxicity of nanoparticles: Inhibition of seed germination and root growth. Environ. Pollut..

[42] Lin D.H., Xing B.S. (2008). Root uptake and phytotoxicity of ZnO nanoparticles. Environ. Sci. Technol..

[43] Gardea-Torresdey J.L., Rico C.M., White J.C. (2014). Trophic transfer, transformation, and impact of engineered nanomaterials in terrestrial environments. Environ. Sci. Technol..

[44] López-Moreno M.L., De La Rosa G., Hernàndez-Viezcas J.A., Peralta-Videa J.R., Gardea-Torresdey J.L. (2010). X-ray absorption spectroscopy (XAS) corroboration of the uptake and storage of CeO_2_ nanoparticles and assessment of their differential toxicity in four edible plant species. J. Agric. Food Chem..

[45] Andersen C.P., King G., Plocher M., Storm M., Pokhrel L.R., Johnson M.G., Rygiewicz P.T. (2016). Germination and early plant development of ten plant species exposed to titanium dioxide and cerium oxide nanoparticles. Environ. Toxicol. Chem..

[46] Slomberg D.L., Schoenfisch M.H. (2012). Silica nanoparticle phytotoxicity to *Arabidopsis thaliana*. Environ. Sci. Technol..

[47] Zhang P., Ma Y., Zhang Z., He X., Zhang J., Guo Z., Tai R., Zhao Y., Chai Z. (2012). Biotransformation of ceria nanoparticles in cucumber plants. ACS Nano.

[48] Zhang Z., He X., Zhang H., Ma Y., Zhang P., Ding Y., Zhao Y. (2011). Uptake and distribution of ceria nanoparticles in cucumber plants. Metallomics.

[49] Layet C., Auffan M., Santaella C., Chevassus-Rosset C., Montes M., Ortet P., Barakat M., Collin B., Legros S., Bravin M.N. (2017). Evidence that soil properties and organic coating drive the phytoavailability of cerium oxide nanoparticles. Environ. Sci. Technol..

[50] Dan Y., Ma X., Zhang W., Liu K. (2016). Single particle ICP-MS method development for the determination of plant uptake and accumulation of CeO_2_ nanoparticles. Anal. Bioanal. Chem..

[51] Zhang P., Ma Y., Liu S., Wang G., Zhang J., He X., Zhang J., Rui Y., Zhang Z. (2017). Phytotoxicity, uptake and transformation of nano-CeO_2_ in sand cultured romaine lettuce. Environ. Pollut..

[52] Zhang W., Dan Y., Shi H., Ma X. (2017). Elucidating the mechanisms for plant uptake and in-planta speciation of cerium in radish (*Raphanus sativus* L.) treated with cerium oxide nanoparticles. J. Environ. Chem. Eng..

[53] Ma Y., Zhang P., Zhang Z., He X., Zhang J., Ding Y., Zhang J., Zheng L., Guo Z., Zhang L. (2015). Where does the transformation of precipitated ceria nanoparticles in hydroponic plants take place?. Environ. Sci. Technol..

[54] Bao D., Oh Z.G., Chen Z. (2016). Characterization of silver nanoparticles internalized by Arabidopsis plants using single particle ICP-MS analysis. Front. Plant Sci..

[55] Corredor E., Testillano P.S., Coronado M.J., Gonzalez-Melendi P., Fernandez-Pacheco R., Marquina C., Ibarra M.R., De La Fuente J.M., Rubiales D., Perez De Luque A. (2009). Nanoparticle penetration and transport in living pumpkin plants: In situ subcellular identification. BMC Plant Biol..

[56] Avellan A., Schwab F., Masion A., Chaurand P., Borschneck D., Vidal V., Rose J., Santaella C., Levard C. (2017). Nanoparticle uptake in plants: Gold nanomaterial localized in roots of Arabidopsis thaliana by X-ray computed nanotomography and hyperspectral imaging. Environ. Sci. Technol..

[57] Geisler–Lee J., Wang Q., Yao Y., Zhang W., Geisler M., Li K., Huang Y., Chen Y., Kolmakov A., Ma X. (2013). Phytotoxicity, accumulation and transport of silver nanoparticles by *Arabidopsis thaliana*. Nanotoxicology.

[58] Ma Y.H., He X., Zhang P., Zhang Z.Y., Guo Z., Tai R.Z., Xu Z.J., Zhang L.J., Ding Y.Y., Zhao Y.L. (2011). Phytotoxicity and biotransformation of La_2_O_3_ nanoparticles in a terrestrial plant cucumber (*Cucumis sativus*). Nanotoxicology.

[59] Ma X., Geiser-Lee M.J., Deng Y., Kolmakov A. (2010). Interactions between engineered nanoparticles (ENPs) and plants: Phytotoxicity, uptake and accumulation. Sci. Total Environ..

[60] Nair R., Varghese S.H., Nair B.G., Maekawa T., Yoshida Y., Kumar D.S. (2010). Nanoparticulate material delivery to plants. Plant Sci..

[61] Lv J.T., Zhang S.Z., Luo L., Zhang J., Yang K., Christie P. (2015). Accumulation, speciation and uptake pathway of ZnO nanoparticles in maize. Environ. Sci. Nano.

[62] Schymura S., Fricke T., Hildebrand H., Franke K. (2017). Elucidating the role of dissolution in CeO_2_ nanoparticle plant uptake by smart radiolabeling. Angew. Chem. Int. Ed..

[63] McCully M. (1995). How do real roots work? (Some new views of root structure). Plant Physiol..

[64] Dan Y., Zhang W., Xue R., Ma X., Stephan C., Shi H. (2015). Characterization of gold nanoparticle uptake by tomato plants using enzymatic extraction followed by single-particle inductively coupled plasma-mass spectrometry analysis. Environ. Sci. Technol..

[65] López-Moreno M.L., de la Rosa G., Hernàndez-Viezcas J.A., Castillo Michel H., Botez C.E., Peralta-Videa J.R., Gardea-Torresdey J.L. (2010). Evidence of the differential biotransformation and genotoxicity of ZnO and CeO_2_ nanoparticles on soybean (*Glycine max*) plants. Environ. Sci. Technol..

[66] Mattiello A., Pošćić F., Musetti R., Giordano C., Vischi M., Filippi A., Bertolini A., Marchiol L. (2015). Evidences of genotoxicity and phytotoxicity in Hordeum vulgare exposed to CeO_2_ and TiO_2_ nanoparticles. Front. Plant Sci..

[67] Shtangeeva I. (2014). Europium and cerium accumulation in wheat and rye seedlings. Water Air Soil Pollut..

[68] Ramos S.J., Dinali G.S., Oliveira C., Martins G.C., Moreira C.G., Siqueira J.O., Guilherme L.R.G. (2016). Rare earth elements in the soil environment. Curr. Pollut. Rep..

[69] Pošćić F., Schat H., Marchiol L. (2017). Cerium negatively impacts the nutritional status in rapeseed. Sci. Total Environ..

[70] Zhao J., Stenzel M.H. (2018). Entry of nanoparticles into cells: The importance of nanoparticle properties. Polym. Chem..

[71] Banerjee K., Pramanik P., Maity A., Joshi D.C., Wani S.H., Krishnan P., Ghorbanpour M., Wani S.H. (2019). Methods of using nanomaterials to plant systems and their delivery to plants (Mode of entry, uptake, translocation, accumulation, biotransformation and barriers). Advances in Phytonanotechnology: From Synthesis to Application.

