# Supplementary material for: Germination and Early Development of Three Spontaneous Plant Species Exposed to Nanoceria (nCeO2) with Different Concentrations and Particle Sizes
Source: Nanomaterials (Basel). 2020 Dec 17;10(12):2534. doi: 10.3390/nano10122534 (PMC7766237; doi:10.3390/nano10122534)
Supplement: Supplementary file 1 [file nanomaterials-10-02534-s001.pdf]

nanomaterials

Article

Germination and Early Development of Three Spontaneous Plant Species Exposed to Nanoceria ($nCeO_2$) with Different Concentrations and Particle Sizes

Daniel Lizzi [1,2], Alessandro Mattiello [1], Barbara Piani [1], Guido Fellet [1], Alessio Adamiano [3] and Luca Marchiol [1,*]

[1] DI4A—Department of Agriculture, Food, Environment and Animal Sciences, University of Udine, Via delle Scienze 206, 33100 Udine, Italy; lizzi.daniel.1@spes.uniud.it (D.L.); alessandro.mattiello@uniud.it (A.M.); barbara.piani@uniud.it (B.P.); guido.fellet@uniud.it (G.F.)

[2] Department of Life Sciences, University of Trieste, Via Licio Giorgieri 10, 34127 Trieste, Italy

[3] Institute of Science and Technology for Ceramics (ISTEC), National Research Council (CNR), Via Granarolo 64, 48018 Faenza, Italy; alessio.adamiano@istec.cnr.it

* Correspondence: marchiol@uniud.it

Table 1. – Theoretical ratio calculated at a hypothetical equal mass of nanoparticles uptaken by plants exposed to nCeO_2 25 nm and 50 nm. The ratio is calculated by dividing the number of *n*CeO_2 25nm by the number of *n*CeO_2 50nm; both numbers are calculated dividing the mass by the estimated the mass of a single nanoparticle (g/g).

Species	Treatment	[Ce] plant ($mg L^{-1}$)	Mass (g)	*n*CeO_2 Ø (nm)	*n*CeO_2 volume (nm^3)	*n*CeO_2 density ($g nm^3$)	*n*CeO_2 mass† (g)	*n*CeO_2‡ (n)	Theoretical ratio§
All	All	es: 200	0.2	25	8181	7.22E-27	5.91E-23	3.39E+21	8.0
			0.2	50	65448	7.22E-27	4.73E-22	4.23E+20	

† single *n*CeO_2; ‡ mass/mass of a single *n*CeO_2; § *n*CeO_2 25nm / *n*CeO_2 50nm;

Table 2. – The observed ratio calculated at the measured mean Ce uptake by the plants exposed to the two nCeO_2 25 nm and 50 nm for the treatments 200 and 2000ppm and the two species *L. flos-cuculi* and *D. tenuifolia*. The ratio is calculated by dividing the number of nCeO_2 25nm by the number of nCeO2 50nm; both numbers are calculated dividing the mass of Ce derived from the measured mean Ce by the estimated the mass of a single nanoparticle (g/g).

Species	Treatment	[Ce] plant ($mg L^{-1}$)	Mass (g)	*n*CeO_2 Ø (nm)	*n*CeO_2 volume (nm^3)	*n*CeO_2 density ($g nm^3$)	*n*CeO_2 mass† (g)	*n*CeO_2‡ (n)	Theoretical ratio§
Lychnis flos-cuculi	200	165	0.165	25	8181	7.22E-27	5.91E-23	2.79E+21	10.3
		128	0.128	50	65448		4.73E-22	2.71E+20	
	2000	1616	1.616	25	8181		5.91E-23	2.74E+22	11.2
		1151	1.151	50	65448		4.73E-22	2.44E+21	
Diplotaxis tenuifolia	200	189	0.189	25	8181	7.22E-27	5.91E-23	3.20E+21	13.3
		114	0.114	50	65448		4.73E-22	2.41E+20	
	2000	1841	1.841	25	8181		5.91E-23	3.12E+22	11.3
		1305	1.305	50	65448		4.73E-22	2.76E+21	

† single *n*CeO_2; ‡ mass/mass of a single *n*CeO_2; § *n*CeO_2 25nm / *n*CeO_2 50nm;
